# P-58. Quantifying the impact of introducing a new adult-focused PCV in the United States

**DOI:** 10.1093/ofid/ofae631.265

**Published:** 2025-01-29

**Authors:** Kevin Bakker, Giulio Meleleo, Oluwaseun Sharomi, Robert Nachbar, Rachel Oidtman

**Affiliations:** Merck & Co., Inc., Rahway, NJ, USA, Philadelphia, Pennsylvania; Wolfram Research, Inc., Champaign, Illinois; Merck & Co., Inc., Lansdale, Pennsylvania; Wolfram Research, Inc., Champaign, Illinois; Merck & Co., Inc., Lansdale, Pennsylvania

## Abstract

**Background:**

In the United States, pneumococcal conjugate vaccines (PCVs) were first recommended for use in children in 2000, and approximately 82% currently receive the full 3+1 schedule. As of May 2024, adults aged 65 years and older are currently recommended to receive PCV15+PPSV23 or PCV20. Approximately 60% of adults have received at least one pneumococcal vaccination. A new investigational PCV, V116, is designed specifically for adults, as it contains 21 serotypes which account for the majority of pneumococcal disease in adults, including 8 unique serotypes not included in any other currently licensed vaccine. We sought to quantify the health impact of the new adult focus vaccine, V116 in comparison to PCV20 in adults.

**Table 1:**
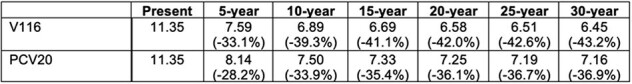
Total IPD incidence per 100,000 (all ages) for each scenario at different time points. Also shown is the percent change from present values.

**Methods:**

We developed a compartmental model to capture pneumococcal carriage transmission in the United States in the presence of age- and serotype-specific pneumococcal vaccines. A dynamic transmission model is preferable as it accounts for serotype replacement and herd protection. We calibrated the model to historical age- and serotype-specific invasive pneumococcal disease (IPD) data in the United States. The model was then used to quantify the epidemiological differences between PCV20 or V116 in 65+ year-olds in the presence of pediatric vaccination. We assumed pediatric vaccination continued at 82% coverage with an 80/20 mix of PCV20/PCV15 and that 57% of adults aged 65+ would have received a PCV in the last 10 years.

**Table 2:**
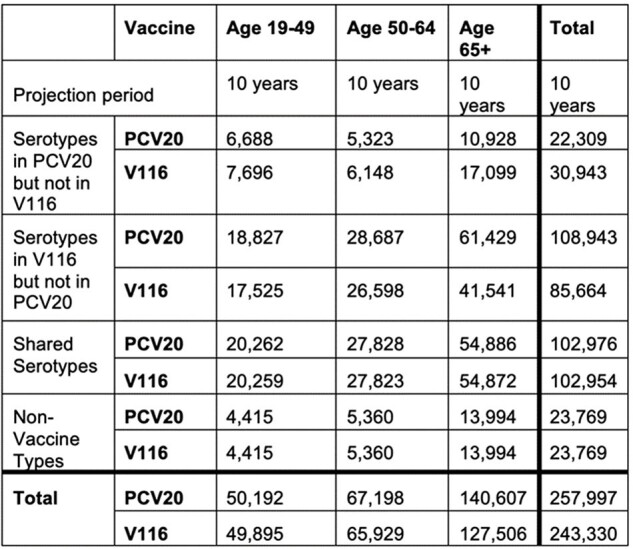
Cumulative IPD cases in a 10-year projection period for V116 and PCV20.

**Results:**

While the introduction and continued use of either PCV20 or V116 led to reductions in IPD incidence, the use of V116 led to fewer overall cases. When compared to current IPD incidence, V116 led to an overall 39.3% reduction across all ages while PCV20 led to a 33.7% reduction after 10 years (Table 1).

**Conclusion:**

The use of V116 in adults combined with continued pediatric PCV vaccination reduced population level IPD incidence by nearly 40% in the United States after only 10 years, which equated to approximately 15,000 fewer cases with V116 than PCV20 (Table 2). While there was a greater number of cases in the serotypes not included in V116 when compared to adult PCV20 use, indirect protection from pediatric vaccination still led to declines in these serotypes from present values.

**Disclosures:**

**Kevin Bakker, PhD**, Merck & Co., Inc.: Grant/Research Support|Merck & Co., Inc.: Stocks/Bonds (Public Company) **Giulio Meleleo, PhD**, Merck & Co., Inc.: Vendor **Oluwaseun Sharomi, MSc, PhD**, Merck & Co., Inc.: Full time employee|Merck & Co., Inc.: Stocks/Bonds (Public Company) **Robert Nachbar, PhD**, Merck & Co., Inc.: US 7,219,020, US 5,292,741|Merck & Co., Inc.: Vendor|Merck & Co., Inc.: Stocks/Bonds (Public Company) **Rachel Oidtman, PhD**, Merck & Co., Inc.: Full time employee|Merck & Co., Inc.: Stocks/Bonds (Public Company)

